# Enhancing Heterogeneous Communication for Foggy Highways Using Vehicular Platoons and SDN

**DOI:** 10.3390/s25030696

**Published:** 2025-01-24

**Authors:** Hafiza Zunera Abdul Sattar, Huma Ghafoor, Insoo Koo

**Affiliations:** 1 School of Electrical Engineering and Computer Science (SEECS), National University of Sciences and Technology (NUST), Islamabad 44000, Pakistan; hsattar.msee21seecs@seecs.edu.pk; 2 Department of Electrical, Electronic and Computer Engineering, University of Ulsan, Ulsan 77024, Republic of Korea

**Keywords:** local controllers (LCs), main controller (MC), platoons, software-defined vehicular network (SDVN), vehicle-to-infrastructure (V2I), vehicle-to-vehicle (V2V)

## Abstract

Establishing a safe and stable routing path for a source–destination pair is necessary regardless of the weather conditions. The reason for this is that vehicles can improve safety on the road by exchanging messages and updating each other on the current conditions of both roads and vehicles. This paper intends to solve the problem of when foggy roads make it difficult for drivers to travel, especially when people encounter emergency situations and have no other option but to drive in foggy weather. Although the literature offers few solutions to the problem, no one has considered integrating software-defined networking into vehicular networks for foggy roads to create an optimal routing path. Moreover, it is of significance to mention that vehicles in adverse weather conditions travel following each other and maintaining a constant safety distance, which leads to the formation of a platoon. Considering this, we propose a heterogeneous communication protocol in a software-defined vehicular network to establish an optimal routing path using platoons on foggy highways. Different cases were tested to show how platoons behave in high connectivity and sparsity, achieving a maximum delivery ratio of 99%, a delay of 2 ms, an overhead of 55%, and an acceptable number of hops compared to reference schemes.

## 1. Introduction

Weather conditions affect driving when people are moving, especially when they need to travel in an emergency situation. Foggy weather in various countries makes it difficult for people to travel safely and reach their destinations on time due to low visibility on the roads. Often, there is no alternative that ensures people will reach their destinations safely and within time. The coordination between vehicles using a periodic exchange of messages to create a safe and stable routing path helps drivers to drive on foggy roads. The issue is that these messages need to be delivered in a shorter time frame with less overhead to prevent accidents and avoid burdening the network with additional overhead. Secondly, it is highly desirable that these messages reach their destination with high success rates.

To ensure timely delivery and lower overhead, roadside units (RSUs) are viewed as an effective way to improve the performance of vehicle-to-vehicle (V2V) communication. The literature [1] has already discussed multiple routing schemes that ensure safe and stable routing paths for both V2V and vehicle-to-infrastructure (V2I) communications in clear weather. However, to implement these protocols in foggy weather, we require new technology that enhances network intelligence and offers flexibility, as fog presents its own challenges regarding loss of signal. To ensure safe movement in adverse weather conditions, we require a software-based controller that handles queries from vehicles and instructs them accordingly. Therefore, our proposed scheme should involve a method that focuses on software-based controllers, and with the consideration of these control messages, it assures timely delivery with minimal overhead during foggy weather. For decades, software-defined networking (SDN) [2] has been considered an emerging technology that enhances vehicular network performance by reducing forwarding delay and network overhead and preventing hop-by-hop flooding during route discovery.

The network is viewed globally by the main controller (MC) of SDNs. However, the routing traffic cannot be controlled by a single MC in this dynamic and challenging network. Therefore, there is a need for local controllers (LCs) that manage network traffic at a lower level than the MC. By managing routing traffic at the local level, these LCs assist the MC, sharing its burden. Hence, our priority is to choose these LCs using a decision tree classifier (DTC), since MC and RSUs (a few as LCs) form a tree-based topology in a vehicular network. These LCs also assist vehicles in finding the optimal routing path. Selecting the optimal path in a foggy environment can be a bit of a challenge, but taking advantage of vehicles that move at an almost constant speed during foggy weather as they follow each other across the low visibility road makes it possible. Vehicles typically follow the driver who is familiar with the area they are passing through at that moment. In a nutshell, vehicles move in platoons to traverse the low-visibility area. Our LCs guide these vehicles to cross any foggy areas on the road by sending periodic alert messages (PAMs).

We use a dedicated short-range communication (DSRC) band for V2V and V2I communications, as it performs well in foggy weather. Moreover, as our scheme integrates SDN technology into vehicular networks, this integration is named software-defined vehicular network (SDVN) [3]. Hence, our aim is to implement SDVN during foggy weather by introducing a MC that maintains a global network view by obtaining locally collected data from LCs. These MC and LCs form the control layer of the SDN technology. They help vehicles moving in the form of a platoon by instructing them about the current visibility conditions and to reduce speed and change lanes using PAMs. The controllers communicate with each other using LTE technology to maintain stability and high coverage range, while V2V and V2I communications use DSRC technology. The key contributions of this paper are as follows:

(i) We introduce SDN for vehicular communications on fog-shrouded highways to find a safe, stable, and optimal routing path between the source platoon and the destination platoon. The path requires vehicular speed and current visibility along with other important factors to allow vehicles to move safely while exchanging PAMs during foggy weather.
(ii) The job of MC is to select the LCs using DTC that reduce delays and overhead by bringing them locally near the vehicles, so that the LCs can periodically monitor the current visibility and instruct the vehicles to move accordingly by altering their speed during low visibility conditions.

The remainder of the paper is organized as follows. Section 2 discusses related work, Section 3 introduces our proposed scheme, Section 4 discusses the results, and Section 5 concludes the article with future directions.

## 2. Related Work

The literature [4] provides very few solutions to overcome the issue of driving in foggy weather by providing safe and stable schemes using various methods, ensuring that drivers reach their destinations safely during foggy weather. Vehicular networks benefit from the use of visual sensors to enhance incidental awareness, ensure safe handling of messages, and enable intelligent vehicle navigation. Providing optimal routing paths in adverse weather conditions with high image quality becomes more challenging due to light absorption and scattering. A three-stage deep network enabled dehazing network (TSDNet) [5] was proposed to improve image quality in foggy environments. Similarly, the efficiency of light-emitting diodes in V2V communication during foggy weather was tested [6,7] using visible light communication. The test was applied up to a visibility distance of 120 m. The results showed that the signal efficiency was high for 20 m. The performance was affected by the decrease in efficiency of these sensors caused by high fog with visibility less than 20 m.

The DSRC standard for both V2V and V2I communications has no loss during foggy weather [8]. The network efficiency of this 75 MHz dedicated band is cheaper compared to the visual sensor solutions in the literature because of a low loss of signal strength. Very little research has been conducted in the literature specifically focused on foggy weather using DSRC, incorporating an examination of all the issues discussed above. In [9], a traffic warning message dissemination system (TWMDS) framework along with a protocol called the reverse routing protocol (RRP) was proposed for disseminating traffic warning messages (TWMs) in VANETs. Colored Petri net models were used to formally model and analyze the interaction behavior of TWMDS, providing a feasibility measure for its development and implementation. This method demonstrated that drivers have the ability to reroute their paths to avoid traffic congestion.

Considering all the discussion above, no one has focused on implementing SDN to solve this adverse weather issue using the DSRC band. SDVN [2,3], provides flexibility and reliability in such a highly dynamic environment by reducing delay and overhead, thus ensuring a high delivery ratio. Ref. [10] proposed a truck platooning system using SDN technology. The framework aimed to provide a practical solution for SDVN that can be leveraged for a wide range of applications, including truck platooning. The efficiency of the SDN-based approach compared to traditional routing approaches was demonstrated through the truck platooning use case, highlighting the advantages of dynamic interface management and the use of SDN controllers for improved connectivity. The authors also mentioned the use of micro-commands for coordinating intra-platoon or inter-platoon maneuvers, such as merge, split, and lane change, which rely on the exchange of beacon safety messages. The study considered only homogeneous networks of vehicles, like truck platooning.

Similarly, another scheme [11] that considers the impact of fog on visibility was evaluated using an intelligent driver model to address this problem. A platoon of 51 vehicles was used to evaluate the model on a 2.2 km ring road for 250 s. According to the results, the model accurately described the traffic with reduced acceleration and deceleration. Moreover, ref. [12] considers that DSRC technology was proposed as a non-SDN approach for foggy weather. The scheme considered the wireless fog warning system (WFWS) for dissemination of warning messages to control sudden braking on foggy roads. This was a non-SDN approach that took a minimum visibility of 50 m. To manage traffic on foggy roads, the authors used broadcasting of warning messages, which resulted in high overhead. To ensure coverage area, a minimum controller selection mechanism was proposed in [13] to reduce the number of controllers. The scheme considered real-time traffic to determine which controller should be switched on for data transmission. In clear weather, the experimental results were carried out, which achieved a high delivery ratio and low latency.

Another scheme [14] was proposed to distribute the load of an SDN controller by another sub-layer of controllers in clear weather to enable vehicles moving at high speeds on highways to access multiple services. Similarly, the SDN schemes were used in the literature to select routing paths in vehicular networks during clear weather. A greedy routing based on graph convolutional networks was proposed in the hybrid SDVN [15]. The SDN controller is responsible for training the decision model globally, and vehicles use this model for routing decisions. For high-speed mobile vehicles, an optimal base station was considered in [16] to reduce the cost of route updates and ensure a seamless handover of vehicles in SDVN. Their experimental results demonstrated a significant improvement in network service quality and handover performance. Clear weather scenarios were also considered in this scheme. The propagation delay was estimated in [17] using a novel computationally efficient solution to estimate the parameters of the channel model. The channel characteristics were well-fitted with the theory results, and the model was practical for characterizing beyond 5G V2V communications, as evidenced by the numerical results. Similar to describing the non-stationary nature of multi-mobility V2X channels, a time-varying acceleration model was proposed in [18] to describe the motion of the communication terminals and scattering clusters. The simulation results validated the utility of the model for identifying V2X channels.

Thus, the literature shows that the existing schemes do not consider forming an optimal routing path using SDN technology in vehicular networks to overcome the issues of high latency, large overhead, and low successful packet delivery on foggy highways. In addition, none of the schemes discussed above considered visibility less than 20 m to accommodate more challenging scenarios, nor implemented SDN technology to improve network performance during foggy weather. Therefore, we aim to implement a SDVN network to resolve the issue of high latency and large overhead in foggy weather while optimizing safe and stable routing paths. DSRC is a standard band dedicated for vehicular communication that can withstand foggy weather. In addition, the efficiency of communication based on DSRC is greater than that of visual sensors. Thus, in order to capitalize on this, we introduce a novel SDVN architecture for foggy weather using a DSRC band. Hence, the use of heterogeneous communications can lead to an optimal routing path with low latency and low overhead. All the schemes discussed above are compared in Table 1.

## 3. Proposed Scheme: SDVN for Foggy Highways

**Figure 1:**
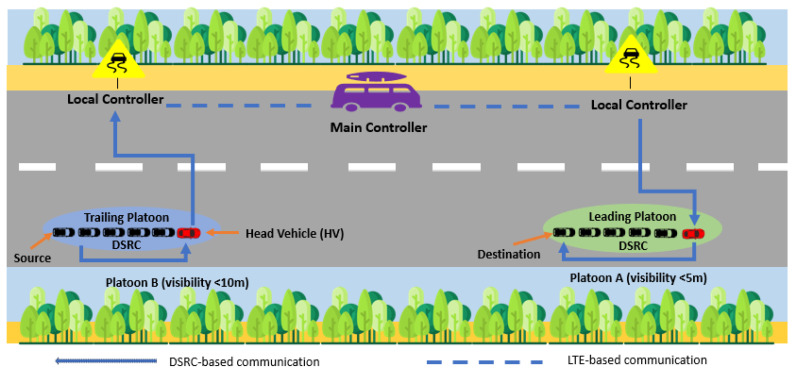
A machine learning SDVN model for foggy highways.

To overcome the adverse weather effect while driving, we propose a platoon-based communication system in SDVN, as shown in Figure 1. The MC is considered a dedicated infrastructure (such as a toll booth, a motorway police office, or a parked motorway police car) in a highway scenario to monitor the overall network traffic. We will consider the city scenario in the near future. The RSUs (such as street lights, cameras, or sign boards) act as LCs to share the network traffic load of a single MC. Not all RSUs are LCs, otherwise our aim of proposing an optimal routing path would fail. Our objective is to reduce delay and overhead in a foggy environment by ensuring connectivity, which is why only a few out of all RSUs serve as LCs. As a result of this division, the MC and LCs are responsible for managing network traffic at both the global and local levels, respectively. DSRC technology is used by vehicles within the platoon to communicate. If inter-platoon communication is not possible using DSRC due to distance, LCs are used as relays, as can be seen in Figure 1.

Considering a foggy environment on roads with very limited visibility, in this work, we consider different cases for handling network traffic with safe and stable communication links. These communication links then form an optimal routing path between any source and destination by exchanging current status information (such as vehicle ID, position (x,y), velocity (vel), current visibility ($vis_{c}$), and current time (*t*)) with each other and with any LC within their communication range. Similarly, the head vehicle (HV) of the platoon is responsible for exchanging the collected data from its platoon to those heads that are within the communication range of this platoon. By doing so, a stable path between platoons is created, which enables any source vehicle in a trailing platoon that is far away from the destination vehicle in a leading platoon to determine the current visibility status at the destination location. In a sequence of platoons traveling along the same route as shown in Figure 1, the leading platoon is the platoon that is at the front, while the platoon that trails the leading platoon is called the trailing platoon.

**Figure 2:**
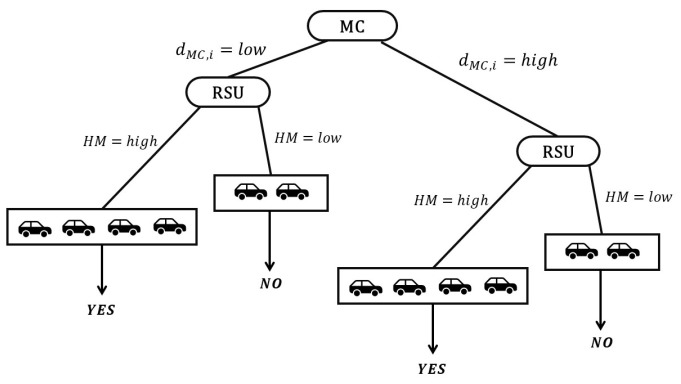
Decision tree for selecting local controllers.

A question may arise for the reader as to who makes the selections for the LCs and who makes the decisions for the HV in a platoon. The answer for both is the MC. We use DTC to locate the LCs at each level on the road. DTC is used because RSUs are placed at various levels on the road, producing a tree-based topology where MC is the parent node, and all remaining RSUs are the child nodes. The parameters we consider are the distance of each RSU from the MC, the number of hello messages received per second to count connectivity, and the number of hops required to reach the MC (see Figure 2). Based on these parameters, we choose the LC with the minimum delay (MD) at each depth of the tree as

(1) $$MD_{controller}=\left(\frac{d_{MC,i}}{\alpha \times speed}+\frac{L_{s}}{R_{MC,i}}+\frac{t}{HM}\right)HC,$$

where $d_{MC,i}$ indicates the Euclidean distance between MC and each *i*th RSU, $L_{s}$ is the packet size, $R_{MC,i}$ is the rate (bps) of each link, *t* is the current time, HM is the amount of hello messages received periodically from vehicles within communication range of each *i*th RSU, HC is the number of hops that are counted to reach the MC, a constant α is used for a balance, and speed is the speed of light since we assume that the communication links between MC and RSUs are LTE-based. Each RSU calculates its $MD_{controller}$ from the MC, which takes into account the distance ($d_{MC,i}$) from the MC, the number of hops (HC) needed to get there, and the density of vehicles at the time *t*. If the $MD_{controller}$ value is lower, the number of hops will be lower, and the density of vehicles will be higher. As a result, the RSU with the lowest $MD_{controller}$ is chosen to be the LC at each layer from the MC. Figure 2 illustrates a tree-based topology where the MC is the parent node and RSUs at various levels from the MC are the child nodes. The RSU positions are exact depictions of their actual location on the highway, as highways do not have RSUs like signboards, streetlights, or cameras located in the same place. From (1), it is evident that HM represents the total number of hello messages that each RSU receives from the vehicles within its communication range. The higher the number of vehicles within an RSU range, the greater the likelihood of it being chosen as the LC.

For the selection of HV, we assume that vehicles that are moving on the road at the same speed and have the same destination can be part of a platoon. The HV vehicle in our scheme can be led by a driver who knows how to drive in critical situations, maintains speed, and has strong psychological attributes. These driver attributes will be added to our experiment as an extension of this paper. In this article, we consider the first on the road in a platoon as the HV. To explain our algorithm in detail, we use an example scenario in which MC has selected all LCs and HVs (see Figure 1). The visibility on the highway becomes low as the platoons proceed towards the foggy region. Figure 1 shows two platoons moving on the road at different speeds and facing different visibility regions. The communication range of an LC encompasses each platoon. PAMs are exchanged between platoons through LCs (if two platoons are not within communication range) and within the platoon directly using DSRC. These PAMs include platoon ID, vehicular ID, (x,y), vel, $vis_{c}$, and *t*. The HV collects the current status (vehicle ID, (x,y), vel, $vis_{c}$, and *t*) of all vehicles within its platoon and forwards this computed information to the nearby LCs. Since LCs are connected directly through LTE, this locally collected information is shared with the MC and with only LCs involved in the optimal routing path (to decrease the periodic exchange of messages). Thus, based on a limited exchange of control packets, which ultimately reduces network overhead, MC creates a global network view. Different cases are considered below to test the validity of our proposed scheme in low-visibility scenarios.

### 3.1. Case (i): High Connectivity—When There Are Platoons on the Highway and They Are in Proximity to the LCs

As can be seen in Figure 1, platoon A enters a low visibility region where HV instructs all its members to slow down their speed. Before it instructs its members, the LC has already sent a PAM to the HV to reduce its speed due to low visibility ahead. Let us assume that a source vehicle in platoon B, at its current position, intends to communicate with the destination vehicle in platoon A to learn about the road conditions (or visibility conditions) at the location where platoon A is moving. The source vehicle sends a request message to its HV first, and then the HV asks its nearby LC (within communication range CR), by sending a packet_in message, to find an optimal routing path to platoon A (see Figure 1). This optimal path is calculated as follows:

(2) $$Path_{optimal}=max\left(PT_{p}\right),$$

where the path-time (PT) is calculated using link-duration (LD). If the link is between platoon to platoon or platoon to LC, it is $LD_{platoon}$, and if it is between LC to LC, it is $LD_{controller}$, such that

(3) $$PT_{p}=min\left(LD_{pc(1,p)},LD_{pc(2,p)},.,LD_{pc(l,p)}\right),$$

where pc represents either platoon or controller, based on the communication technology, *l* represents the total number of relay nodes, which is usually LCs, but there is a possibility that HVs and members can become relays between two communicating platoons (see next case). p=1,2,…,P represents all possible paths *P* between two communicating platoons. The maximum PT is determined by using LD to calculate several links along a single path between the source and destination platoons. The minimum LD is chosen for each path because ignoring it could result in the path breaking. Additionally, there are several paths between source and destination. We select LD with the maximum PT from all these minimums. Hence, $LD_{pc}$ indicates the minimum delay at the data layer of the SDVN protocol when platoons communicate, which is calculated as

(4) $$LD_{pc,ij}=\frac{CR_{\max}\pm d_{ij}}{vel_{ij}}\times \frac{vis_{c}}{vis_{\max}}.$$

The link with minimum $LD_{pc}$ is selected as the next hop for establishing an optimal routing path between the source and the destination in two different platoons. When this pc is the controller, the $vel_{ij}$ is equal to the speed of light because the communication is LTE-based. When this pc is the platoon, the $vel_{ij}$ is the speed between two vehicles or platoons (if vehicles inside the platoon are moving at the same speed). $vis_{c}$ is between 5 m and 10 m at a certain location, ±shows both directions of traffic, and $vis_{max}$ is the maximum visibility (50 m) we considered. Equation (4) shows how vehicles connect with each other based on current foggy conditions. Now, we explain using Equation (5) how vehicles in platoon B adjust their speed by maintaining a safe distance between each other when they are aware of lower visibility conditions on the next patch of road.

(5) $$v_{f_{t}}=v_{f_{t-1}}+\frac{v_{f_{t-1}}-v_{i}}{t^{2}}-\frac{{\mathrm{vis}}_{c}}{t_{s}},$$

where $v_{f_{t}}$ represents the final velocity (current velocity) a vehicle adjusts to in *t* seconds in order to prevent a collision and $v_{f_{t-1}}$ represents the final velocity in previous time. Meanwhile, $v_{i}$ denotes the vehicle’s initial velocity in previous time (before acceleration), and $t_{s}$ stands for the simulation (current) time.

### 3.2. Case (ii): When the Previous Platoon Is Moving Faster than the Leading Platoon

**Figure 3:**
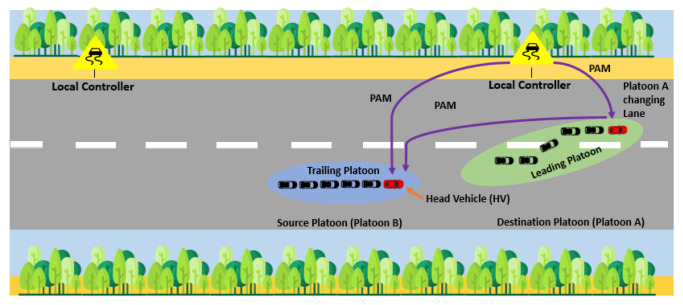
A lane change scenario in a foggy SDVN.

In this case, we consider that when platoon B is moving at a higher speed than platoon A, it reaches the low-visibility region earlier. Platoon A and LCs are constantly sending PAMs to platoon B, letting it know of the low visibility ahead. If, for some reason, platoon B does not respond appropriately, LCs may ask platoon A to change lane immediately as shown in Figure 3. Thus, to prevent collisions between vehicles, Equation (5) involves maintaining a safety distance depending on the speed between vehicles. Equation (5) is applicable only if ${\mathrm{vis}}_{c}$ < 20 m. This means that the scheme does not allow vehicles to change lanes when visibility is <5 m. To keep Equation (5) valid, it is crucial to keep in mind that the new velocity will soon become 0 if visibility drops below 5 m. According to our algorithm, all vehicles are required to stop when visibility is less than 5 m. If platoon B responds to these PAMs, there is no need for platoon A to change lanes.

### 3.3. Case (iii): Low Connectivity: When the Network Is Sparse, a Platoon May Find It Difficult to Locate an LC Within Its Range

**Figure 4:**
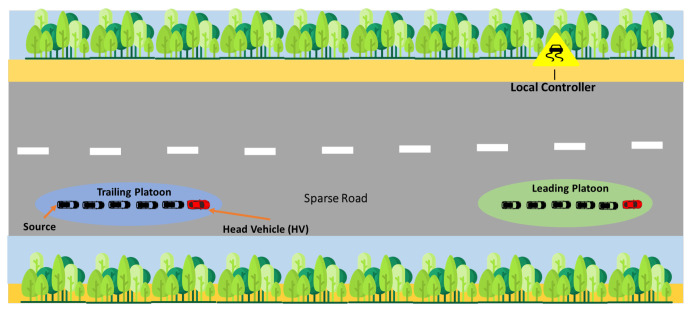
A sparse network scenario in a foggy SDVN.

There is a possibility in such a highly dynamic network with such critical weather conditions that no platoon finds another platoon or LC in its vicinity, as can be seen in Figure 4. For instance, if a querying platoon is looking for a leading node to inform it about the current visibility in its proceeding path, it becomes challenging for a source platoon to find a relay to reach the destination platoon. In this scenario, the HV stores and carries the packet until it reaches the communication range of some LCs or other nodes on the highway. This may increase the time to convey any message in the network, but it is better to store the packet rather than drop it. To sum up, we ensure safe and stable driving during foggy weather with the help of SDN controllers that ensure connectivity and form stable communication links by instructing platoons according to visibility conditions, leading to optimal routing paths for exchanging urgent messages. We will demonstrate this using simulation results for each case in the following section.

## 4. Performance Evaluation

We evaluated the proposed scheme by optimizing the routing path in foggy weather on a 10 km highway by introducing platoons that move according to controller instructions and adjust their speed based on current visibility conditions. Five distinct platoons of vehicles were deployed along the highway. The speed of five cars in a platoon was varied, ranging from 5 to 10 m/s, with vehicles maintaining a minimum distance of 20 m and a maximum intra-platoon distance of 200 m. Different speeds were assigned to each platoon in a range of 5 to 20 m/s. The platoons changed their lane when the inter-platoon distance was less than 20 m. These platoons and vehicles were assigned unique identifiers to distinguish them. As our objective was to find an optimal routing path from the source platoon to the destination platoon on a foggy highway, we considered $CR_{max}$ = 200 m for V2V communication and 500 m for V2I (vehicle to LC) communication. There were one MC, 10 RSUs, and five LCs in this topology, and $L_{s}$ = 16 bytes, $R_{MC,i}$ = 10 Mbps, and α = 2/3 [19]. For V2V and V2I communication, DSRC was used operating in a 5.9 GHz frequency band. Direct, low-latency communication between vehicles was made possible by DSRC, which supports critical messages and is immune to fog [8,20]. The communication model between the LCs and MC used the LTE cellular model, providing wide coverage for efficient data exchange, thereby ensuring reliable coordination between them.

In a highway scenario, both small-scale and large-scale fading can affect the radio channel for V2V and V2I communications. It is common for there to be no line of sight between a transmitter and a receiver. The network performance may be affected by faded signals as the receiver signal is made up of various multipath components [21]. The Nakagami distribution is used to describe the statistical characteristics of both small-scale and large-scale fading, which is why we chose the Nakagami model for radio channel characteristics. In MATLAB-22b, a real-world scenario of highway road and vehicles was created. The platoons followed a straight path on the highway. OpenFlow [22] was chosen as the underlying protocol because it is widely accepted and flexible in routing strategies. The message passing mechanisms in MATLAB have been modified to replicate OpenFlow message exchanges. The performance was evaluated to assess the effectiveness of the proposed scheme by considering the following parameters. In order to compare our proposed cases, we considered [10,11] as reference schemes. The former considered the truck platooning system to instruct trucks about road conditions by providing beacon safety messages during clear weather, and the latter considered an intelligent driver model to handle visibility (minimum of 30 m) on a ring road. We simulated this scheme [11] to determine the following metrics to achieve a perfect match with our scheme. Furthermore, as mentioned in section I, there is no scheme in the literature that addresses SDVN in foggy weather, so we selected these two schemes that have a partial match to our proposed scheme.

- Packet delivery ratio (PDR)
- Average end-to-end delay
- Average number of hops
- Routing overhead ratio (RoR)

### 4.1. Packet Delivery Ratio (PDR)

**Figure 5:**
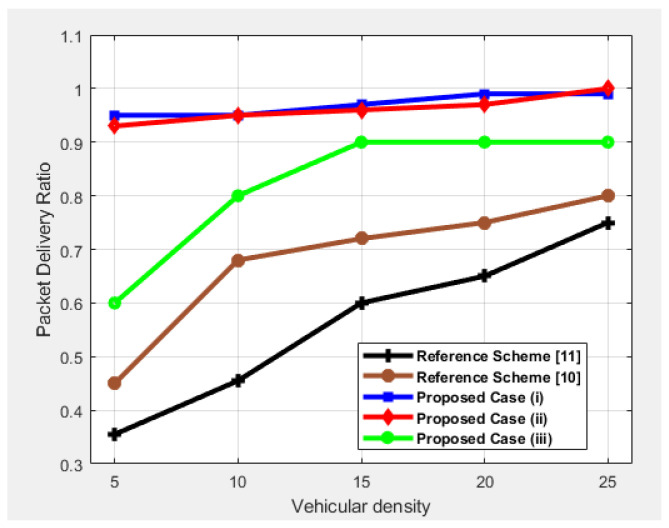
Performance comparison in terms of PDR as a function of vehicular density.

PDR is defined as the ratio of packets received by the destination platoon to the packets sent by the source platoon. We tested the PDR as a function of vehicle density, as shown in Figure 5, and found that with increasing vehicle density, the PDR increases. Furthermore, Figure 5 shows all of our cases of high and low connectivity in terms of PDR. We observed an almost identical pattern of increase in PDR with the increasing number of vehicles. However, case (*i*) and (ii) show high PDR in comparison to sparse network conditions because in these cases, connectivity is high. Case (ii) shows a slight decrease due to the increase in speed of the trailing platoon when vehicles are low, but it shows the maximum delivery ratio due to high connectivity. Case (iii) also requires a relay to demonstrate the connection between nodes, which is why its performance is lower than that of the other two cases. When the density of the vehicular network increases from 5 to 10 in case (iii), packet delivery is impeded because the increased distance between platoons occasionally prevents the packets from reaching their destination, resulting in reduced PDR. This shows that the two communicating platoons did not establish a link using LCs. The improvement in the PDR can be attributed to the consideration of LCs in the path when increasing the number of platoons in the network. Our proposed cases introduce the concept of vehicles communicating with LCs when there are no neighboring vehicles within their communication range. This ensures successful packet delivery, achieving 99% PDR, as all packets are effectively transmitted from the source platoon to the destination platoon, regardless of vehicular density in case (*i*) and case (ii). Regarding case (ii), because the platoons are moving in both lanes, it thereby further enhances the PDR. Figure 5 also shows that the reference scheme [10] exhibits better performance due to SDN than the reference scheme [11], but its performance is inferior to our scheme due to the consideration of high-height vehicles. However, the reference scheme [11] shows poor performance because it does not consider SDN and focuses more on broadcasting messages rather than ensuring successful delivery.

### 4.2. Average End-to-End Delay

**Figure 6:**
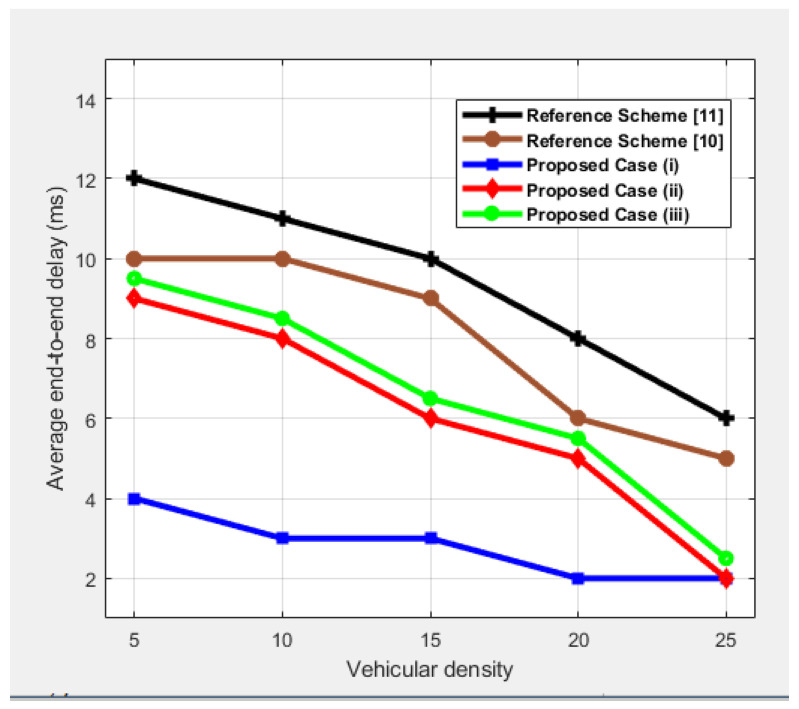
Performance comparison in terms of end-to-end delay as a function of vehicular density.

Average end-to-end delay is defined as the total average time a source platoon takes to deliver a message to the destination platoon. Figure 6 shows the comparison of average delay in terms of vehicular density. It can be clearly seen that all cases show the same pattern in which delay decreases with an increase in vehicular density. This is logical because, as vehicle density increases in case (*i*), communication becomes more efficient, resulting in reduced delay, since vehicles are consistently within the communication range, facilitating straightforward packet delivery from the source to the destination platoons. Conversely, for the other two proposed cases, a slight increase in delay is observed. This occurs because when vehicles cannot immediately find nearby counterparts due to low visibility within their communication range, they pause momentarily, allowing for other vehicles to come within range. If no suitable vehicles are found, they initiate communication with the LCs if they are within the proximity of the platoons. This brief pause introduces a minor delay, but it ensures that all packets are successfully transmitted, maintaining efficient and reliable communication, albeit with a small delay trade-off. Similarly, the reference schemes exhibit high delays due to the lack of LCs that manage traffic, resulting in the continuous broadcast of warning messages.

### 4.3. Average Number of Hops

**Figure 7:**
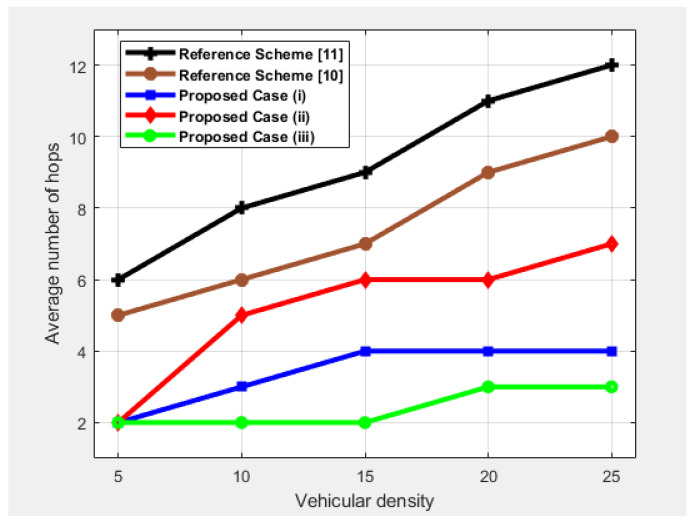
Performance comparison in terms of average number of hops as a function of vehicular density.

The average number of hops is measured as the average number of hop counts in establishing an optimal, safe, and stable routing path from source to destination. Figure 7 shows the comparison of the average number of hops as a function of vehicular density. Depending on different cases and different visibility scenarios, the number of hops increases with increasing density for each source–destination pair. For case (iii), since the source stores and carries the packet until it comes within a range of any other node, the number of hops is less. For the other two cases, the increase in the number of hops occurs because the data packets travel through more vehicles or LCs to reach their destination. Moreover, when vehicular density increases, the hops in a sparse network become fixed. Because LCs are directly connected using LTE, the same number of hops is used for the remaining number of vehicles. The number of hops required to deliver packets in case (ii) is high because in this case, the vehicles change lanes, so it takes time for the whole platoon to become part of the second lane. Additionally, they ensure connectivity, which increases hops. Regarding this lane change scenario, it involves both LCs and HV forwarding PAMs to the trailing platoon to reduce its speed. Thus, in comparison to case (*i*), two links for the same destination are formed to avoid accidents in low visibility scenarios. However, case (*i*) has fewer hops in sparse conditions as LCs are used to convey messages to leading platoons. However, it becomes stable for the rest of the vehicle density due to increased connectivity. Figure 7 also shows that the number of hops for the reference schemes increases with the density of vehicles. This is due to the lack of LCs-based V2I communication in low-visibility scenarios.

### 4.4. Routing Overhead Ratio (RoR)

**Figure 8:**
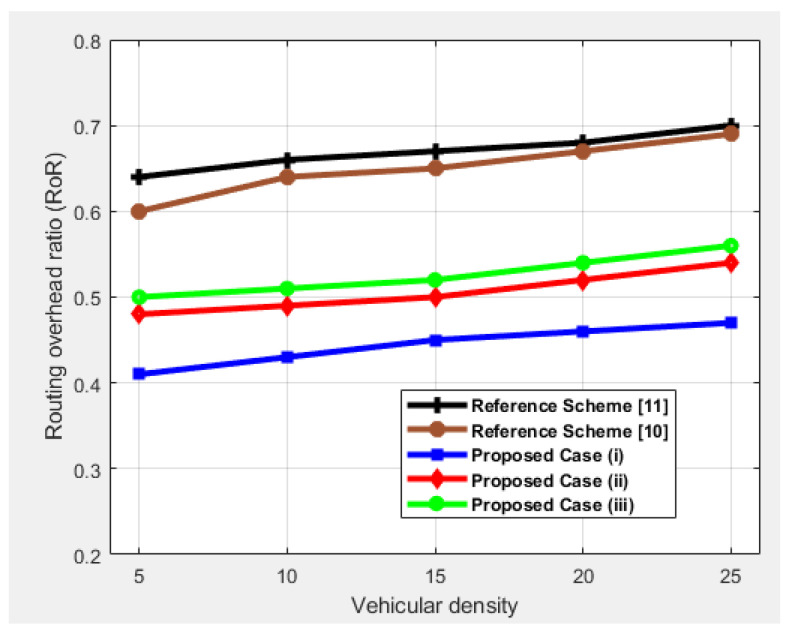
Performance comparison in terms of routing overhead ratio as a function of vehicular density.

RoR is the ratio between the number of control packets and the entire network packets. The pattern for all the cases shows an increase in overhead due to an increase in vehicular density as can be seen in Figure 8. Because the number of nodes increases, the overhead increases to ensure connectivity. Case (iii) shows high overhead due to finding a relay node within range. It needs to send control packets until it reaches any LC or HV. Case (*i*) shows high connectivity, which means less overhead as compared to the other two cases. In case (ii), the RoR is higher due to the need to exchange more packets in order to avoid collisions. If there are no SDN local controllers, then the exchange of warning messages is in the form of broadcasting, which leads to high overhead, as can be seen in the reference schemes. To sum up, we achieved high performance in all four parameters in comparison to the reference schemes, thus ensuring that the use of SDN in vehicular networks during foggy weather makes it possible for vehicles to travel safely by finding an optimal routing path.

**Figure 9:**
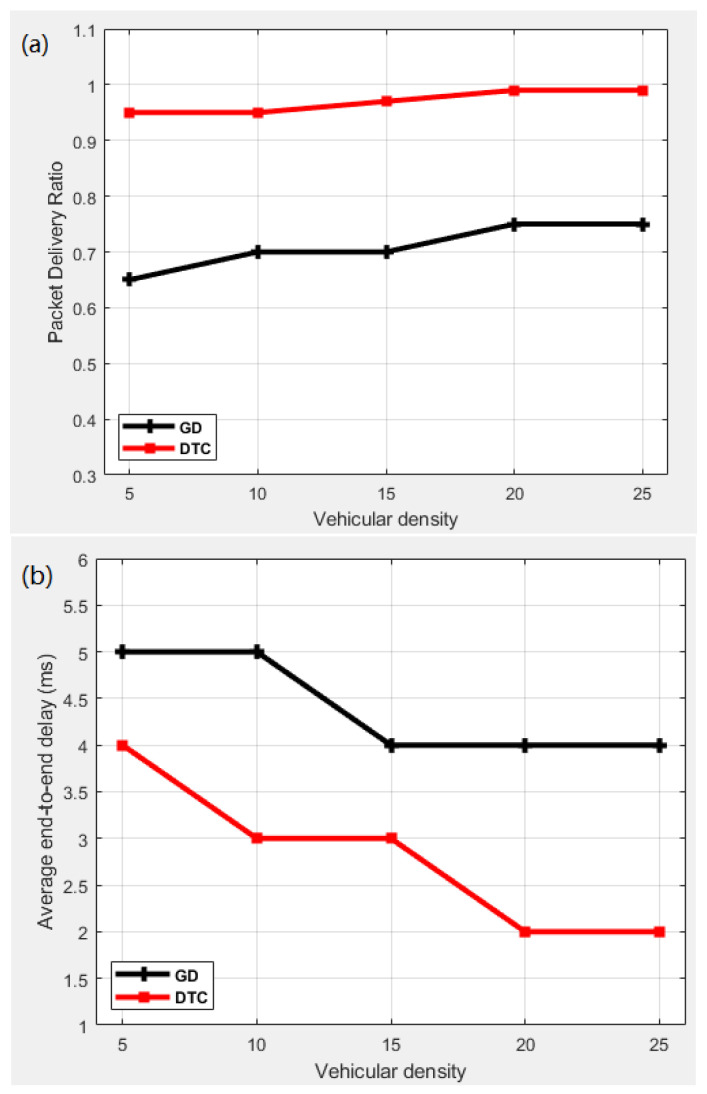

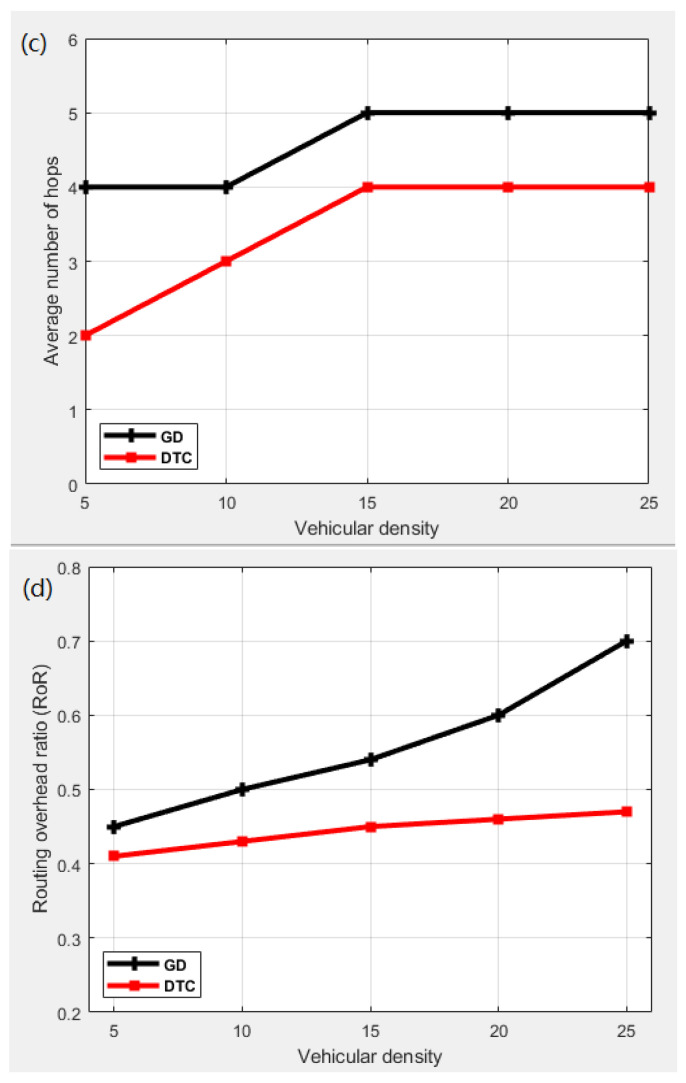
Performance comparison for DTC and GD on highway scenario in terms of (**a**) PDR, (**b**) average delay, (**c**) number of hops, and (**d**) RoR.

To justify our preference for DTC in our scheme, we examined another set of comparisons for all four parameters in Figure 9. The comparison between the performance of DTC and the gradient descent (GD) algorithm is made to assess their ability to predict LCs based on the number of vehicles. Both models were trained and implemented in our code to select LCs. The dataset was produced by randomly generating vehicle data. The features included vehicle speed, vehicle density, and the positions of RSUs to determine which LCs should be selected. Due to their position, different RSUs have different road traffic. Traffic signals always have a high density when they are red; therefore, position plays a significant role in selecting the LCs. Likewise, speed is crucial as it reflects how long a vehicle stays within the communication range of an RSU. Table 2 illustrates the use of multiple metrics to evaluate the effectiveness of the models. After calculating the accuracy of both models, we observed that DTC achieved 90% accuracy while GD achieved 82.2% accuracy. Hence, DTC was more preferred in our scheme to choose LCs than GD for this reason. DTC is able to handle non-linear relationships among features with minimal computational requirements, which is typically the case in highly dynamic environments like VANETs. Figure 9 illustrates the high connectivity situation of platoons on highways where the patterns for both algorithms are almost the same. However, the DTC exhibits superior performance in each PDR, delay, hops, and RoR, which demonstrates its high accuracy when compared to GD.

## 5. Conclusions

We proposed a heterogeneous (DSRC and LTE) communication in SDVN to address the issues faced by drivers during foggy weather. The main controller (MC) selects local controllers (LCs) using a decision tree classifier to assist vehicular platoons in sending periodic alert messages to find an optimal routing path. This safe and stable path takes into account vehicular speed and current visibility in a specific location to communicate the message about speed reduction or lane change in the shortest time possible. The LCs help platoons to make timely decisions to reduce their speed when visibility is getting worse and when there is a leading platoon moving at low speed. The head vehicle of a platoon collects data from within the platoon and updates the LCs with the locally collected data, enabling the MC to construct a global network view. The results demonstrated the validity of this stable communication in terms of delivery ratio, end-to-end delay, average number of hops, and routing overhead ratio compared to two reference schemes. Our future work considers the city model and psychological characteristics of drivers to enhance the performance of the network for heavy fog that results in visibility of less than 5 m.

## Figures and Tables

**Table 1:** Comparison of related schemes.

Protocols	Consider Foggy Weather?	Consider SDN?	Use DSRC Band?	Use Heterogeneous Communication?	Select Optimal Path?
[5]	✓	×	×	×	×
[6]	✓	×	×	×	×
[7]	✓	×	×	×	×
[9]	×	×	✓	×	✓
[10]	×	✓	✓	×	✓
[11]	✓	×	×	×	×
[12]	✓	×	✓	×	×
[13]	×	✓	✓	×	✓
[14]	×	✓	✓	×	✓
[15]	×	✓	✓	×	✓
[16]	×	✓	✓	×	✓
[17]	×	×	✓	×	×
[18]	×	×	✓	×	×

**Table 2:** Performance metrics for different classifiers.

	Accuracy	Precision	Recall	F1-Score
Gradient Descent	82.2%	86.0%	83.0%	84.5%
Decision Tree Classifier	90%	94.1%	88.9%	91.4%

## Data Availability

No new data were created or analyzed in this study. Data sharing is not applicable to this article.
